# Activity of *N*-Acetylcysteine Alone and in Combination with Colistin against Pseudomonas aeruginosa Biofilms and Transcriptomic Response to *N*-Acetylcysteine Exposure

**DOI:** 10.1128/spectrum.01006-22

**Published:** 2022-06-23

**Authors:** Felice Valzano, Selene Rebecca Boncompagni, Maria Micieli, Tiziana Di Maggio, Vincenzo Di Pilato, Lorenzo Colombini, Francesco Santoro, Gianni Pozzi, Gian Maria Rossolini, Lucia Pallecchi

**Affiliations:** a Department of Medical Biotechnologies, University of Siena, Siena, Italy; b Department of Experimental and Clinical Medicine, University of Florence, Florence, Italy; c Department of Surgical Sciences and Integrated Diagnostics, University of Genoa, Genoa, Italy; d Laboratory of Molecular Microbiology and Biotechnology, Department of Medical Biotechnologies, University of Siena, Siena, Italy; e Clinical Microbiology and Virology Unit, Careggi University Hospital, Florence, Italy; University of Guelph

**Keywords:** *N*-acetylcysteine, *Pseudomonas aeruginosa*, biofilms, colistin, cystic fibrosis, synergism, transcriptomic response

## Abstract

Chronic colonization by Pseudomonas aeruginosa is critical in cystic fibrosis (CF) and other chronic lung diseases, contributing to disease progression. Biofilm growth and a propensity to evolve multidrug resistance phenotypes drastically limit the available therapeutic options. In this perspective, there has been growing interest in evaluating combination therapies, especially for drugs that can be administered by nebulization, which allows high drug concentrations to be reached at the site of infections while limiting systemic toxicity. Here, we investigated the potential antibiofilm activity of *N*-acetylcysteine (NAC) alone and in combination with colistin against a panel of P. aeruginosa strains (most of which are from CF patients) and the transcriptomic response of a P. aeruginosa CF strain to NAC exposure. NAC alone (8,000 mg/L) showed a limited and strain-dependent antibiofilm activity. Nonetheless, a relevant antibiofilm synergism of NAC-colistin combinations (NAC at 8,000 mg/L plus colistin at 2 to 32 mg/L) was observed with all strains. Synergism was also confirmed with the artificial sputum medium model. RNA sequencing of NAC-exposed planktonic cultures revealed that NAC (8,000 mg/L) mainly induced (i) a Zn^2+^ starvation response (known to induce attenuation of P. aeruginosa virulence), (ii) downregulation of genes of the denitrification apparatus, and (iii) downregulation of flagellar biosynthesis pathway. NAC-mediated inhibition of P. aeruginosa denitrification pathway and flagellum-mediated motility were confirmed experimentally. These findings suggested that NAC-colistin combinations might contribute to the management of biofilm-associated P. aeruginosa lung infections. NAC might also have a role in reducing P. aeruginosa virulence, which could be relevant in the very early stages of lung colonization.

**IMPORTANCE** Pseudomonas aeruginosa biofilm-related chronic lung colonization contributes to cystic fibrosis (CF) disease progression. Colistin is often a last-resort antibiotic for the treatment of such P. aeruginosa infections, and it has been increasingly used in CF, especially by nebulization. *N*-acetylcysteine (NAC) is a mucolytic agent with antioxidant activity, commonly administered with antibiotics for the treatment of lower respiratory tract infections. Here, we show that NAC potentiated colistin activity against *in vitro* biofilms models of P. aeruginosa strains, with both drugs tested at the high concentrations achievable after nebulization. In addition, we report the first transcriptomic data on the P. aeruginosa response to NAC exposure.

## INTRODUCTION

Pseudomonas aeruginosa is a leading pathogen infecting the airways of patients affected by cystic fibrosis (CF) and other chronic lung diseases (e.g., chronic obstructive pulmonary disease and non-CF bronchiectasis) (1). Once established in the CF airways, P. aeruginosa develops into chronic infections and generally persists indefinitely, contributing to frequent exacerbations, decline of pulmonary function, and higher rates of mortality (1, 2). Chronic infections by P. aeruginosa in CF lungs are associated with adaptive changes of the pathogen, such as conversion to a mucoid phenotype, switching to the biofilm mode of growth, and acquisition of antibiotic resistance (3). Cumulative exposure to antibiotics during treatment causes dissemination of multidrug-resistant (MDR) P. aeruginosa strains, leading to the ineffectiveness of the antibiotic therapy and consequently worse clinical outcomes (3).

Colistin is among the last-resort agents for the treatment of P. aeruginosa infections caused by MDR strains, with the advantage of being also administrable by nebulization, which allows the achieving of high lung concentrations while reducing systemic toxicity (4). In this perspective, inhaled colistin has been increasingly used for the treatment of difficult-to-treat respiratory tract infections, especially those related to biofilm formation (5).

*N*-acetylcysteine (NAC) is a mucolytic agent commonly administered with antibiotics for the treatment of lower respiratory tract infections, which has been demonstrated to exert also antimicrobial and antibiofilm activity against relevant respiratory pathogens (6–8). Recently, a potent *in vitro* antibiofilm synergism of NAC-colistin combinations was demonstrated against colistin-susceptible and colistin-resistant Acinetobacter baumannii and Stenotrophomonas maltophilia strains (9, 10).

NAC has been demonstrated to exert several heterogeneous biological activities (whose molecular bases have not always been clearly elucidated) and has recently been under extensive investigation for potential clinical applications beyond the approved therapeutic usage as an antidote in acetaminophen (paracetamol) overdose and as a mucolytic (11). Overall, NAC can act as a direct or indirect antioxidant, due to the ability of the free thiol group to react with reactive oxygen and nitrogen species and by constituting a precursor of intracellular glutathione (11). In addition, NAC can bind transition and heavy metal ions and act as a reducing agent of protein sulfhydryl groups involved in intracellular redox homeostasis (11). Despite several studies that have addressed the biological effects of NAC on planktonic and biofilm bacterial cultures (8), to the best of our knowledge, no data on bacterial transcriptomic response to NAC exposure have been reported so far.

In this study, we investigated the *in vitro* antibiofilm activities of NAC alone and in combination with colistin (at the high concentrations achievable by the inhalation route of administration) (8, 12) against a panel of P. aeruginosa strains (most of which are from CF patients) representative of different phenotypes (in terms of mucoidy, antimicrobial susceptibility pattern, and O type) and multilocus sequence type (MLST) genotypes. In addition, we provided original data on the transcriptomic response of P. aeruginosa planktonic cultures to NAC exposure.

## RESULTS AND DISCUSSION

### Activity of NAC alone against preformed biofilm.

The antibiofilm activity of NAC alone was tested with 17 P. aeruginosa strains (Table 1), of which 15 were from CF patients, using the Nunc-TSP lid system.

**TABLE 1:** Features of the 17 P. aeruginosa strains included in this study

Strain	yr of isolation	Phenotype	Origin^*a*^	ST^*b*^	O type	Resistance pattern^*c*^	MIC (mg/L)^*d*^	
							CST	NAC
PAO1	1954	Nonmucoid	Wound	ST549	O5	Wild type	2	64,000
Z33	2005	Nonmucoid	CF	ST235	O11	CP^r^, FQ^r^, AG^r^	1	16,000
Z34	2006	Nonmucoid	CF	ST17	O1	CB^r^, CP^r^, FQ^r^, AG^r^	2	64,000
Z35	2006	Nonmucoid	CF	ST235	O11		1	16,000
Z152	2013	Mucoid	CF	ST155	O6	CB^r^, FQ^r^, AG^r^	2	8,000
Z154	2016	Mucoid	CF	ST412	O6	CP^r^, FQ^r^, AG^r^	2	16,000
M1	2002	Mucoid	CF	ST155	O6	CB^r^, CP^r^, FQ^r^, AG^r^	2	16,000
M4	2005	Mucoid	CF	ST155	O6	CB^r^, CP^r^, FQ^r^, AG^r^	2	32,000
M7	2005	Mucoid	CF	ST253	O10	AG^r^	2	64,000
M13	2000	Mucoid	CF	ST274	O3	CB^r^, CP^r^, AG^r^	1	32,000
M19	2006	Mucoid	CF	ST3509	O7		1	64,000
M25	2002	Mucoid	CF	ST235	O11		2	16,000
M32	2006	Mucoid	CF	ST235	O11		2	16,000
M42	2007	Mucoid	CF	ST2437	O6	CB^r^, CP^r^, FQ^r^, AG^r^	2	32,000
FC237	2007	Nonmucoid	CF	ST365	O3	CB^r^, FQ^r^, AG^r^, CST^r^	512	64,000
FC238	2007	Nonmucoid	CF	ST910	O6	CB^r^, CST^r^	8	64,000
FZ99	2018	Nonmucoid	RTI_ICU_	ST111	O12	CB^r^, CP^r^, FQ^r^, AG^r^, CST^r^	4	64,000

a CF, cystic fibrosis; RTI_ICU_, respiratory tract infection in intensive care unit.

b According to the MLST Pasteur scheme.

c CB^r^, resistance to carbapenems (imipenem and meropenem); CP^r^, resistance to cephems (ceftazidime and cefepime); FQ^r^, resistance to fluoroquinolones (ciprofloxacin); AG^r^, resistance to aminoglycosides (amikacin and gentamicin); CST^r^, resistance to colistin.

d CST, colistin; NAC, *N*-acetylcysteine.

**FIG 1:**
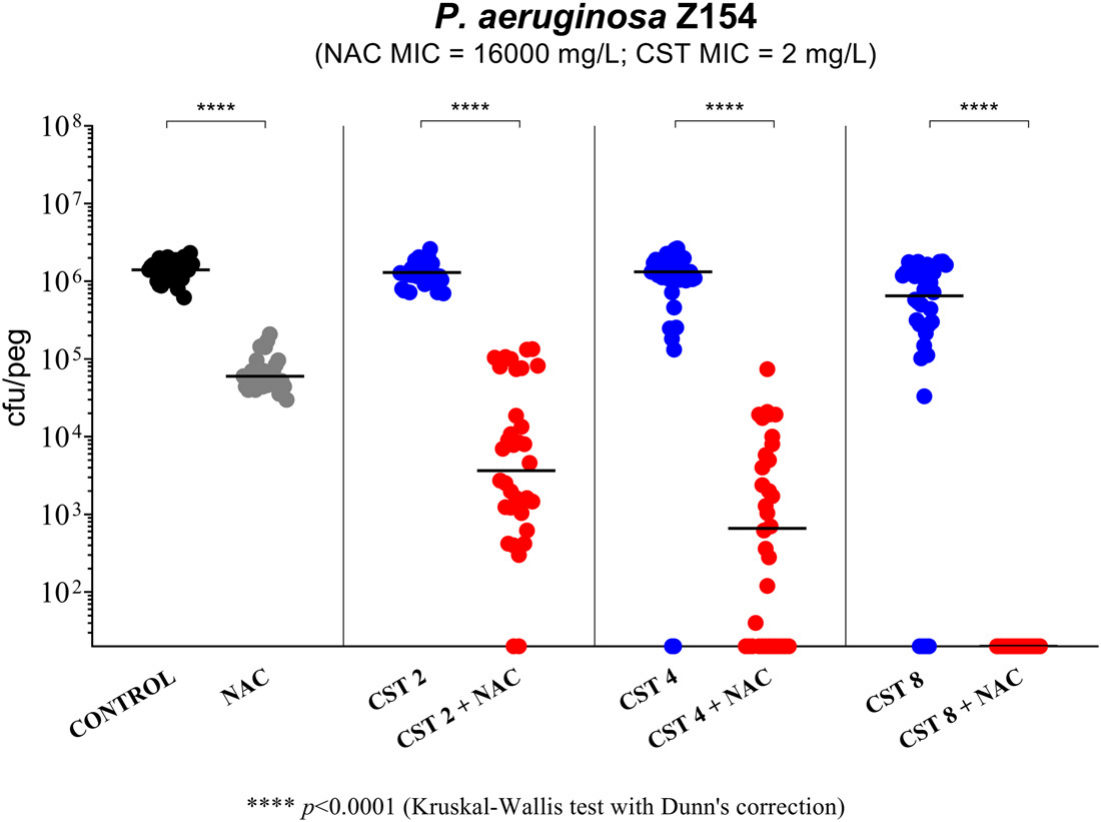
Antibiofilm activity of *N*-acetylcysteine (NAC) at 8,000 mg/L, colistin (CST), and NAC-CST combinations against P. aeruginosa Z154 in the Nunc-TSP lid system. A relevant potentiation of colistin antibiofilm activity was observed with all NAC-CST combinations tested. CST 2, colistin at 2 mg/L; CST 4, colistin at 4 mg/L; CST 8, colistin at 8 mg/L. Biofilms not exposed to NAC or CST represent the control. Black lines indicate median values. The *x* axis is set at the limit of detection (20 CFU/peg).

**FIG 2:**
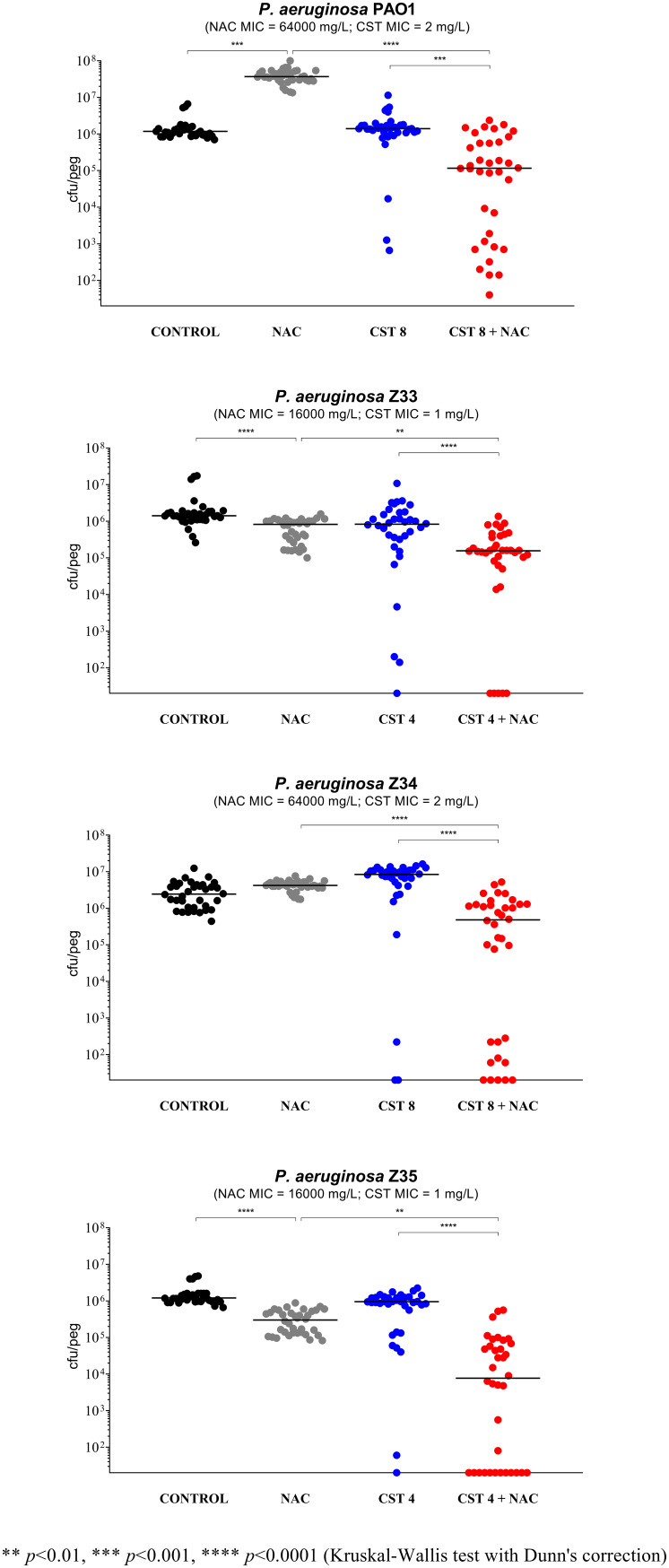
Antibiofilm activity of *N*-acetylcysteine (NAC) at 8,000 mg/L, colistin (CST), and NAC-CST combinations against P. aeruginosa PAO1 and three colistin-susceptible nonmucoid strains in the Nunc-TSP lid system. A potentiation by NAC of colistin antibiofilm activity was observed with all tested strains. CST 4, colistin 4 mg/L; CST 8, colistin 8 mg/L. Biofilms not exposed to NAC or CST represented the control. Black lines indicate median values. The *x* axis is set at the limit of detection (20 CFU/peg).

**FIG 3:**
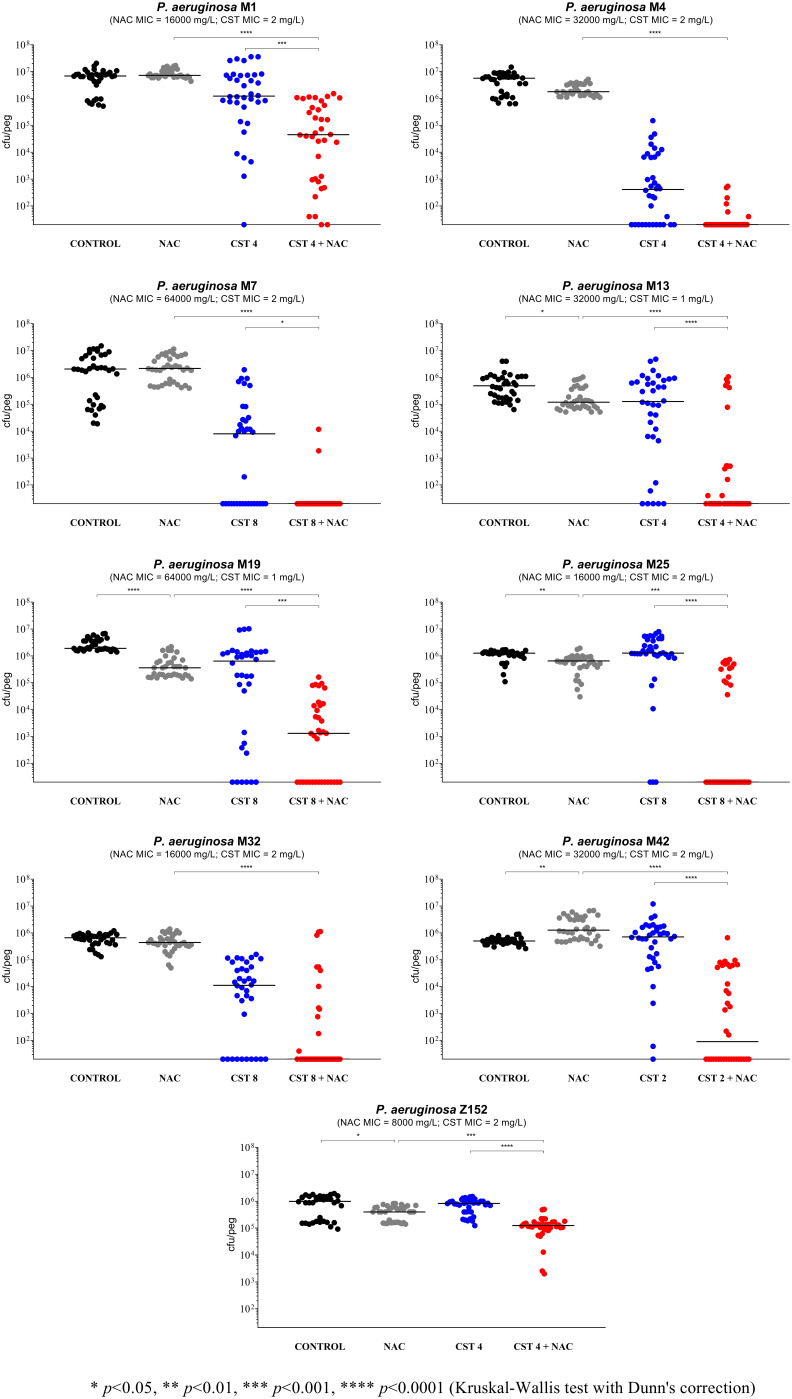
Antibiofilm activity of *N*-acetylcysteine (NAC) at 8,000 mg/L, colistin (CST), and NAC-CST combinations against nine colistin-susceptible mucoid P. aeruginosa strains in the Nunc-TSP lid system. A potentiation by NAC of colistin antibiofilm activity was observed with all tested strains, although in two cases, statistical significance was not achieved (i.e., strains M4 and M32). CST 2, colistin at 2 mg/L; CST 4, colistin at 4 mg/L; CST 8, colistin at 8 mg/L. Biofilms not exposed to NAC or CST represent the control. Black lines indicate median values. The *x* axis is set at the limit of detection (20 CFU/peg).

**FIG 4:**
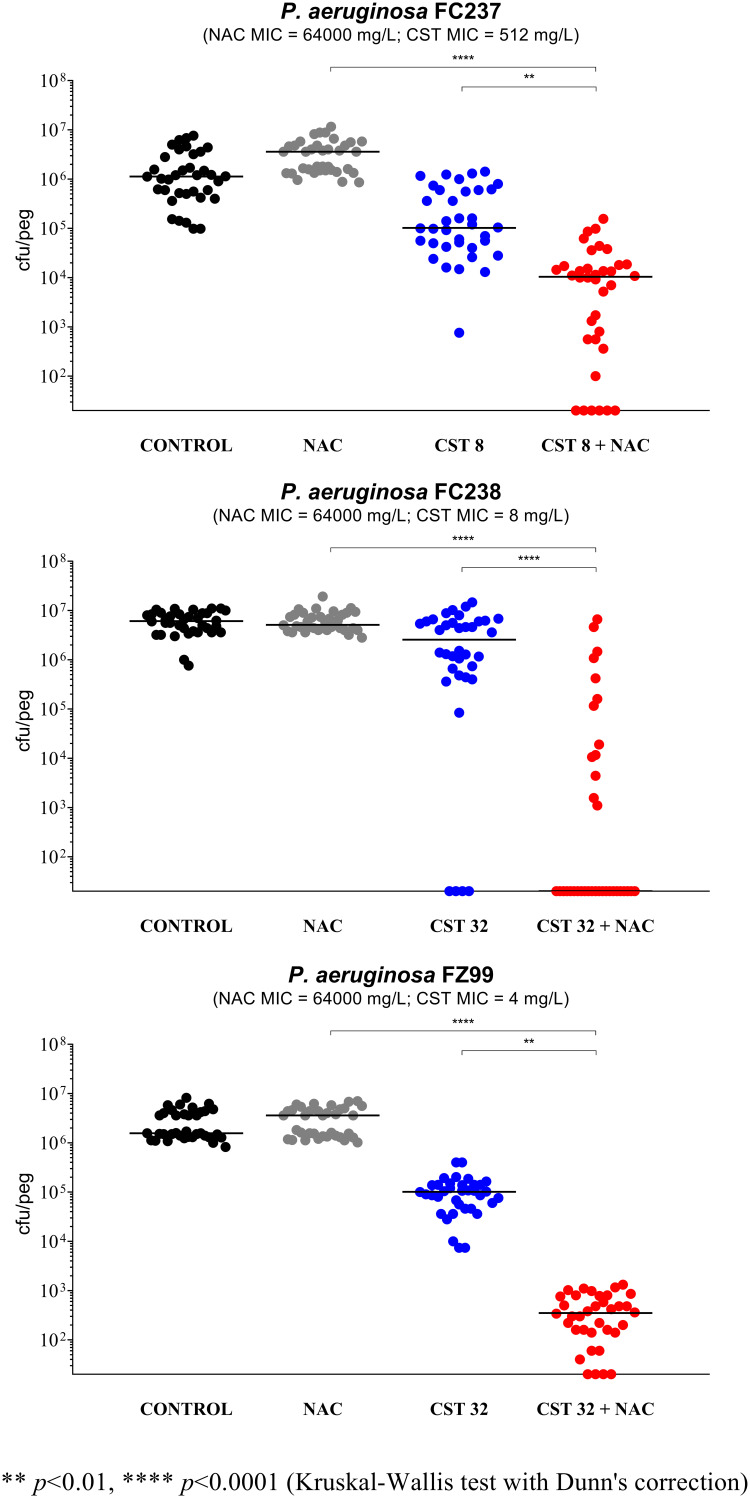
Antibiofilm activity of *N*-acetylcysteine (NAC) at 8,000 mg/L, colistin (CST), and NAC-CST combinations against three colistin-resistant nonmucoid P. aeruginosa strains in the Nunc-TSP lid system. A potentiation by NAC of colistin antibiofilm activity was observed with all tested strains. CST 8, colistin at 8 mg/L; CST 32, colistin at 32 mg/L. Biofilms not exposed to NAC or CST represent the control. Black lines indicate median values. The *x* axis is set at the limit of detection (20 CFU/peg).

NAC at 8,000 mg/L (i.e., a high concentration achievable after inhalation) showed limited and strain-dependent activity (Fig. 1 to 4). In particular, major effects were observed with P. aeruginosa Z154 (i.e., decrease of >1 log CFU/peg compared to the control) (Fig. 1) and P. aeruginosa PAO1 (i.e., increase of >1 log CFU/peg compared to the control) (Fig. 2). With an additional 7 strains, a very slight but statistically significant activity was observed (i.e., <0.5 log CFU/peg compared to the control), resulting in biofilm reduction in six cases (i.e., P. aeruginosa Z33, Z35, Z152, M13, M19, and M25) and biofilm increase in the remaining one (i.e., P. aeruginosa M42) (Fig. 2 and 3).

Overall, these results indicated that inhaled NAC alone might not have major effects on P. aeruginosa biofilms already established in the lung and that the response to NAC was not related to phenotypic or genotypic features. The few previous studies that have addressed the activity of NAC against preformed P. aeruginosa biofilms have reported similar results (i.e., usually limited and strain-dependent effects), although a direct comparison of data is not straightforward due to different methodological approaches (e.g., different biofilm models and different NAC concentrations tested) and the low number of strains often tested in such studies (i.e., usually reference strains) (8, 13, 14). This study provided a wider picture on this topic by investigating a panel of characterized P. aeruginosa strains using a standardized *in vitro* biofilm model and *in vivo* achievable NAC concentrations. Interestingly, NAC alone (at the concentration used in this study and the same biofilm model) was recently shown to exert relevant activity against preformed biofilms of two relevant CF pathogens, namely, S. maltophilia and Burkholderia cepacia complex (BCC) (7). The reasons for such a diverse response of P. aeruginosa compared to S. maltophilia and BCC should deserve further attention, because they could possibly help identifying critical targets in the complex biofilm environments, to be used for the implementation of new antibiofilm strategies.

### Activity of NAC-colistin combinations against preformed biofilms.

P. aeruginosa Z154 (a mucoid, MDR, colistin-susceptible CF strain) was first used to test the potential antibiofilm synergism of NAC at 8,000 mg/L plus diverse colistin concentrations. As shown in Fig. 1, a relevant synergism was observed already with colistin at 2 mg/L (i.e., the colistin MIC for the tested strain), with a dose-dependent effect at increasing colistin concentrations, and complete biofilm eradication was achieved with the combination of NAC at 8,000 mg/L plus colistin at 8 mg/L (Fig. 1).

The remaining 16 strains were initially tested with the combination of NAC at 8,000 mg/L plus colistin at 8 mg/L. In order to detect a potential synergism, the concentration of colistin was then modified for strains forming biofilms highly susceptible to colistin (*n* = 7) or particularly resistant (*n* = 2) (Fig. 2 to 4). Overall, a relevant synergism of NAC-colistin combinations was observed with all tested strains (including the three colistin-resistant ones), although in two cases (i.e., P. aeruginosa M4 and M32), statistical significance was not achieved (Fig. 2 to 4). These latter strains were also tested with lower colistin concentrations (i.e., 2 and 4 mg/L, respectively), but synergism was not observed (data not shown). Concerning the synergism observed with the three colistin-resistant strains (Fig. 4), it is interesting to note that with strain FC237 (nonmucoid, MDR), an important decrease in viable biofilm cells was observed with a combination including a colistin concentration much lower than the colistin MIC for this strain (i.e., 1/64 MIC) (Fig. 4).

Overall, these data demonstrated that NAC could potentiate colistin activity against preformed biofilms of colistin-susceptible and colistin-resistant P. aeruginosa strains, regardless of the mucoid/nonmucoid phenotype, the resistance pattern, and the ST and O type. Present findings are consistent with the previously observed antibiofilm synergism of NAC-colistin combinations against colistin-susceptible and colistin-resistant strains of A. baumannii and S. maltophilia (9, 10). Further studies with a higher number of P. aeruginosa clinical isolates, especially with a colistin-resistant phenotype, are encouraged.

### Activity of NAC-colistin combinations in the ASM biofilm model.

Two P. aeruginosa CF strains exhibiting different phenotypes were selected for susceptibility assays with the artificial sputum medium (ASM) biofilm model: P. aeruginosa Z34 (nonmucoid, MDR, ST17, O1) and P. aeruginosa Z154 (mucoid, MDR, ST412, O6). Biofilms were grown in ASM, in order to mimic the P. aeruginosa biofilm environmental conditions experienced in the CF mucus. Preformed biofilms were then challenged in the same medium with NAC-colistin combinations.

**FIG 5:**
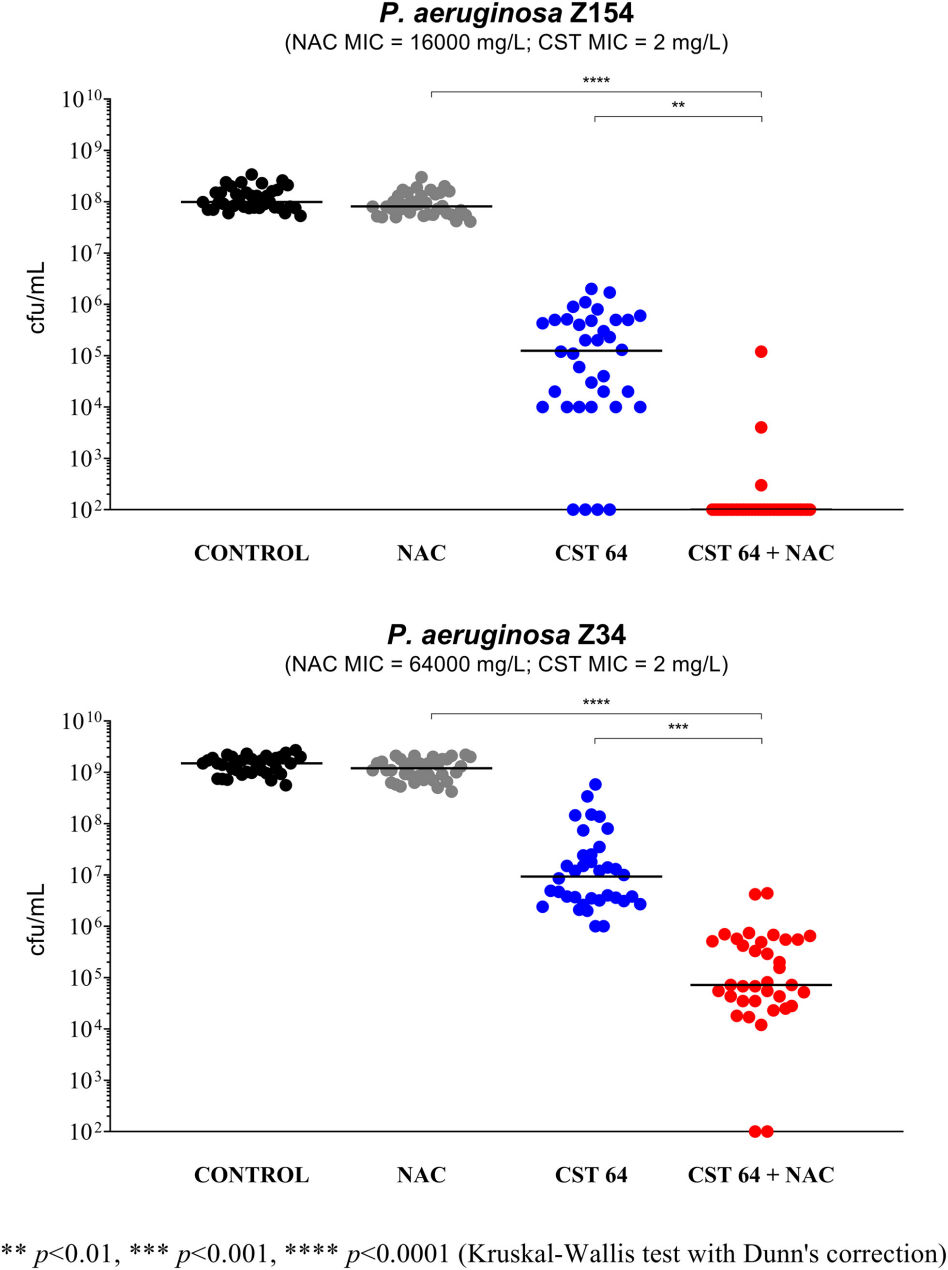
Antibiofilm activity of *N*-acetylcysteine (NAC) at 8,000 mg/L, colistin at 64 mg/L (CST 64), and the NAC-CST combination against P. aeruginosa Z154 and P. aeruginosa Z34 in the ASM biofilm model. A potentiation by NAC of colistin antibiofilm activity was observed with both strains. Biofilms not exposed to NAC or CST represent the control. Black lines indicate median values. The *x* axis is set at the limit of detection (100 CFU/mL).

As shown in Fig. 5, a clear synergism of NAC at 8,000 mg/L in combination with colistin at 64 mg/L was observed with both strains (Fig. 5). Compared to the experiments performed with the Nunc-TSP lid system, the concentration of colistin that allowed observation of a synergism was much higher (i.e., 32× the MIC), possibly due to colistin strong ionic interactions with ASM components (e.g., extracellular DNA and mucin) (15). Indeed, preliminary experiments carried out with lower colistin concentrations did not show either colistin antibiofilm activity or synergism with NAC (data not shown). In addition, the antibiofilm activity of NAC alone observed against P. aeruginosa Z154 in the Nunc-TSP lid system was not observed in the ASM model (Fig. 5), confirming that the efficacy of NAC alone against preformed P. aeruginosa biofilms could be limited *in vivo*.

Overall, these data demonstrated that the antibiofilm synergism of NAC-colistin combinations against P. aeruginosa strains is preserved also under the environmental conditions mimicking the CF mucus, which is promising for clinical applications. Furthermore, the lower susceptibility to colistin of P. aeruginosa biofilms in the ASM model compared to biofilm susceptibility in standard media observed in this study is consistent with what was previously reported with P. aeruginosa (16).

### Transcriptomic response of P. aeruginosa Z154 to NAC exposure.

P. aeruginosa Z154 (i.e., colistin-susceptible CF strain, mucoid, MDR, ST412, O6) was selected for investigating the transcriptome response of planktonic cultures to NAC exposure (i.e., NAC at 8,000 mg/L). A total of 66 differentially expressed genes (DEGs) were identified (adjusted *P* value of <0.05 with 99% confidence interval [CI]), of which 46 were upregulated and 20 downregulated compared to the control (Table 2).

**TABLE 2:** DEGs in P. aeruginosa Z154 planktonic cultures exposed to 8,000 mg/L NAC compared to control

DEG	Locus tag in P. aeruginosa strain			Gene	Product (function)^*a*^	Zur regulon	Adjusted *P* value	Log_2_ fold change
	Z154	PAO1	UCBPP-PA14					
Upregulated	IS492_10415	PA0781	PA14_54180	*znuD*	TBDR ZnuD (zinc uptake)	+	4.6E−36	1.9
	IS492_17070	PA1922	PA14_39650	*cirA*	TBDR CirA (iron and zinc uptake)	+	0.0E+00	2.4
	IS492_17075	PA1923	PA14_39640		Cobaltochelatase subunit CobN-like (cobalamin biosynthesis)	+	7.9E−36	1.9
	IS492_17080	PA1924	PA14_39630	*exbD*	ExbD proton channel family protein (energy support for TBDR, cotranscribed with PA1922)	+	1.7E−03	0.6
	IS492_17085	PA1925	PA14_39620		Hypothetical protein (unknown function, DUF2149 domain-containing protein)	+	7.5E−06	0.8
	IS492_19940	PA2437	PA14_33110		HflC family modulator of membrane FtsH protease	+	5.1E−06	0.8
	IS492_19945	PA2438	PA14_33080		HflC modulator of membrane FtsH protease	+	7.0E−03	0.6
	IS492_19950	PA2439	PA14_33070	*hflK*	HflK family modulator of membrane FtsH protease	+	6.5E−03	0.6
	IS492_23615	PA2911	PA14_26420		TBDR (possibly involved in zinc uptake)	+	7.6E−03	0.6
	IS492_27310	PA3600	PA14_17710	*rpmJ2*	Zinc-independent paralog type B 50S ribosomal protein L36	+	2.0E−16	1.3
	IS492_27315	PA3601	PA14_17700	*rpmE2*	Zinc-independent paralog type B 50S ribosomal protein L31	+	1.2E−04	0.7
	IS492_29825	PA4063	PA14_11320		Zinc SBP (zinc uptake)	+	7.0E−41	2.0
	IS492_29830	PA4064	PA14_11310		Zinc ABC transporter, ATP-binding protein (zinc uptake)	+	4.2E−08	0.9
	IS492_29835	PA4065	PA14_11290		Zinc ABC transporter, permease (zinc uptake)	+	4.9E−13	1.2
	IS492_29840	PA4066	PA14_11280		Zinc SBP (zinc uptake)	+	8.5E−05	0.7
	IS492_06220	PA4834	PA14_63910	*cntI*	Pseudopaline transport plasma membrane protein CntI (zinc uptake)	+	6.1E−05	0.7
	IS492_06215	PA4835	PA14_63920	*cntM*	Pseudopaline biosynthesis dehydrogenase CntM (zinc uptake)	+	8.1E−26	1.7
	IS492_06210	PA4836	PA14_63940	*cntL*	Pseudopaline biosynthesis enzyme CntL (zinc uptake)	+	9.3E−39	2.0
	IS492_06205	PA4837	PA14_63960	*cntO*	Pseudopaline transport outer membrane protein CntO (zinc uptake)	+	0.0E+00	2.5
	IS492_06200	PA4838	PA14_63970		Hypothetical membrane protein	+	8.0E−04	0.7
	IS492_31595	PA5498	PA14_72550	*znuA*	Zinc soluble binding protein ZnuA (zinc uptake)	+	9.0E−08	0.9
	IS492_31600	PA5499	PA14_72560	*zur*	Transcriptional regulator for zinc homeostasis	+	5.3E−10	1.0
	IS492_31605	PA5500	PA14_72580	*znuC*	Zinc ABC transporter, ATP-binding protein ZnuC (zinc uptake)	+	1.2E−07	0.9
	IS492_31610	PA5501	PA14_72590	*znuB*	Zinc ABC transporter, ZnuB permease (zinc uptake)	+	1.9E−03	0.6
	IS492_31780	PA5534	PA14_73000		Hypothetical protein (unknown function, DUF1826 domain-containing protein)	+	9.8E−23	1.5
	IS492_31785	PA5535	PA14_73010	*zigA*	Zinc metallochaperone GTPase ZigA	+	5.9E−42	2.1
	IS492_31790	PA5536	PA14_73020	*dksA2*	Zinc-independent paralog of RNA polymerase-binding protein DksA	+	2.4E−23	1.5
	IS492_31800	PA5538	PA14_73040	*amiA*	*N*-acetylmuramoyl-l-alanine amidase (splitting of septal peptidoglycan during cell division)	+	1.3E−08	1.0
	IS492_31805	PA5539	PA14_73050	*folE2*	Zinc-independent paralog of GTP-cyclohydrolase FolE (folate biosynthesis)	+	4.5E−28	1.7
	IS492_31810	PA5540	PA14_73060	*cam*	γ-Carbonic anhydrase (reversible hydration of carbon dioxide)	+	1.5E−24	1.6
	IS492_31815	PA5541	PA14_73070	*pyrC2*	Zinc-independent paralog of dihydroorotase PyrC (pyrimidine biosynthesis)	+	3.1E−09	1.0
	IS492_02205	PA0433	PA14_05630		Hypothetical protein (unknown function, DUF2946 domain-containing protein)		1.3E−03	0.7
	IS492_02210	PA0434	PA14_05640		TBDR for which the siderophore has not been identified		1.5E−28	1.7
	IS492_02430	PA0478	PA14_06250	*fiuC*	GNAT family *N*-acetyltransferase (release of iron from desferrichrome in the cytoplasm)		3.9E−06	0.8
	IS492_10765	PA0848	PA14_53300	*ahpB*	AhpC-like alkylhydroperoxide reductase (oxidative stress response and cell redox homeostasis)		3.9E−16	1.3
	IS492_17945	PA2100	ND^*b*^	*mdrR2*	Transcriptional regulator, regulatory partner of MdrR1 (regulator of efflux systems)		6.3E−05	0.7
	IS492_17950	PA2101	ND		Conserved hypothetical protein (EamA-like transporter family)		1.7E−26	1.7
	IS492_17955	PA2102	ND		Hypothetical protein (unknown function, Mov34/MPN/PAD-1 family protein)		5.7E−13	1.2
	IS492_17960	PA2103	ND	*moeB*	Probable molybdopterin biosynthesis protein MoeB (ubiquitin-like modifier-activating activity)		7.5E−06	0.8
	IS492_25770	PA3287	PA14_21530		Ankyrin repeat domain-containing protein (unknown function)		1.9E−04	0.7
	IS492_27305	PA3599	PA14_17720		Probable transcriptional regulator		5.2E−12	1.1
	IS492_28275	PA3784	PA14_15130		Hypothetical protein (unknown function)		1.4E−05	0.8
	IS492_28280	PA3785	PA14_15120		Copper chaperone PCu(A)C		8.6E−07	0.9
	IS492_28305	PA3790	PA14_15070		TBDR copper receptor OprC (copper uptake)		1.0E−03	0.6
	IS492_06715	PA4739	PA14_62690		Hypothetical protein (unknown function, BON domain-containing protein)		9.8E−03	0.6
	IS492_31510	PA5481	PA14_72360		Hypothetical periplasmic protein (inhibitor of vertebrate lysozyme)		3.9E−04	0.7

Downregulated	IS492_00850	PA0164	PA14_02050		γ-Glutamyltransferase family protein		8.0E−04	−0.6
	IS492_02660	PA0524	PA14_06830	*norB*	Nitric oxide reductase subunit NorB (denitrification)		3.9E−03	−0.6
	IS492_02685	PA0529	PA14_06890		Hypothetical protein (unknown function, MOSC domain-containing protein)		2.0E−05	−0.7
	IS492_02690	PA0530	PA14_06900		Probable class III pyridoxal phosphate-dependent aminotransferase (diverse metabolic pathways)		5.7E−05	−0.8
	IS492_02695	PA0531	PA14_06920		Aspartate aminotransferase family protein		4.7E−03	−0.6
	IS492_12670	PA1101	PA14_50140	*fliF*	Flagellar M-ring protein FliF (motility)		5.7E−05	−0.7
	IS492_12855	PA1136	PA14_49700		Probable transcriptional regulator		1.5E−12	−1.1
	IS492_12860	PA1137	PA14_49690		Oxidoreductase zinc-binding dehydrogenase family protein (protection from oxidative stress)		0.0E+00	−2.3
	IS492_14625	PA1453	PA14_45660	*flhF*	Flagellar biosynthesis protein FlhF (motility)		7.6E−03	−0.6
	IS492_19230	PA2298	PA14_34900		Probable oxidoreductase		4.9E−05	−0.7
	IS492_19235	PA2299	PA14_34880		Probable transcriptional regulator		3.2E−04	−0.7
	IS492_26340	PA3391	PA14_20230	*nosR*	Regulatory protein NosR (denitrification)		3.2E−04	−0.6
	IS492_26345	PA3392	PA14_20200	*nosZ*	Nitrous oxide reductase (denitrification)		4.1E−05	−0.8
	IS492_26895	PA3519	PA14_18810		Iron-containing redox enzyme family protein		2.8E−05	−0.3
	IS492_26920	PA3523	PA14_18760	*mexP*	Resistance-nodulation-cell division (RND) efflux membrane fusion protein		3.2E−03	−0.2
	IS492_27180	PA3574	PA14_18080	*nalD*	Transcriptional regulator NalD (second repressor of MexAB-OprM)		1.5E−19	−1.3
	IS492_27185	PA3574a	PA14_18070	*copZ*	Copper chaperone CopZ (copper efflux)		9.1E−11	−1.0
	IS492_27760	PA3690	PA14_16660		Heavy metal-translocating P-type ATPase (efflux)		1.1E−08	−1.0
	IS492_28975	PA3920	PA14_13170	*copA*	Copper-translocating P-type ATPase CopA1 (copper efflux)		1.2E−27	−1.2
	IS492_04870	PA5100	PA14_67350	*hutU*	Urocanate hydratase (histidine catabolic process)		4.0E−04	−0.6

a TBDR, TonB-dependent receptor; SBP, soluble binding protein; ABC, ATP-binding cassette. Protein functions were inferred from the literature and PseudoCAP (https://www.Pseudomonas.com/pseudocap).

b ND, not determined.

Analysis of DEGs revealed that NAC mainly acted as Zn^2+^ chelator, inducing a strong Zn^2+^ starvation response. DEGs associated with such response were consistent with data reported in previous studies addressing zinc homeostasis in P. aeruginosa and other bacteria (Table 2) (17–22). In particular, 31 of the 46 upregulated DEGs belonged to the *zur* regulon and are known to be activated in response to Zn^2+^ starvation (Table 2) (17–22). Such genes mainly included operons involved in zinc uptake (e.g., the PA4063-PA4064-PA4065-PA4066 operon, *cntOLMI* operon, and *znuABC* operon) and genes encoding zinc-independent paralogs of cellular proteins (i.e., type B 50S ribosomal proteins L31 and L36, RNA polymerase-binding protein DksA2, adn GTP-cyclohydrolase FolE2) (Table 2) (17–23). Upregulated DEGs belonging to the *zur* regulon also included genes encoding an *N*-acetylmuramoyl-l-alanine amidase (AmiA, involved in splitting of septal peptidoglycan during cell division), a γ-carbonic anhydrase (Cam, involved in reversible hydration of carbon dioxide and important for growth under low-CO_2_ conditions), and three modulators of the membrane FtsH protease (i.e., HflC and HflK family modulators) (Table 2). The membrane FtsH zinc-dependent protease is required for the expression of diverse unrelated phenotypes (e.g., swimming and twitching motility, biofilm formation, autolysis, production of secondary metabolites, maintenance of plasma membrane integrity by degrading misfolded proteins), and it has been recently demonstrated to represent an important virulence factor in P. aeruginosa clone C (23). HflC and HflK family modulators interact with FtsH at the level of the plasma membrane, usually with an inhibitory effect (23). The NAC-mediated effects on the phenotypes related to FtsH would deserve further attention.

The remaining 15 upregulated DEGs included genes encoding a recently described transcriptional regulator, PA2100 (also named MdrR2) (24), an AhpC-like alkyl hydroperoxide reductase (involved in protection from oxidative stress) (25), and proteins possibly involved in copper and iron uptake (Table 2).

MdrR2, together with MdrR1, has been demonstrated to repress the *mexAB-oprM* operon (independently from the MexR repressor), activate the EmrAB efflux pump, and indirectly inhibit biofilm formation (Table 2) (24). The effect of NAC on the MdrR1-MdrR2 dual-regulation system should be further investigated. Nonetheless, a previous study aimed at investigating the potential antagonism of high NAC concentrations (i.e., as those tested in this study) on the activity of the major classes of antibiotics used in the clinical practice, did not show major effects (with the exception of carbapenems, due to a chemical instability of carbapenems in the presence of NAC) (26), suggesting that the activation of the EmrAB efflux could not be relevant or circumvented by compensatory mechanisms.

Analysis of downregulated DEGs identified genes involved in denitrification, in particular *norB* (encoding the nitric oxide reductase subunit NorB), *nosR* (encoding the regulatory protein NosR), and *nosZ* (encoding the nitrous oxide reductase NosZ) (Table 2). These data suggested that NAC might affect P. aeruginosa anaerobic respiration (which is crucial in the deeper biofilm layers and in the CF mucus) (27), because the nitric oxide reductase NorBC and the regulatory protein NosR have been recently demonstrated to constitute the nucleus of the denitrification protein network (28). NAC-mediated inhibition of the P. aeruginosa denitrification pathway might be implicated in the observed antibiofilm synergism of the NAC-colistin combination. Indeed, colistin has been demonstrated to exert increased antibiofilm activity against P. aeruginosa under anaerobic conditions, possibly due to a lower ability to implement the tolerance mechanism (e.g., lipopolysaccharide [LPS] modification) because of the low metabolism accompanying anaerobic growth (29). In this perspective, the inhibition of anaerobic respiration by NAC would further inhibit a P. aeruginosa adaptive response to colistin toxicity. This could be particularly relevant in P. aeruginosa biofilm in the CF mucus, where the anoxic conditions of biofilm cells are related not only to the position of the bacteria within the biofilm (i.e., anoxic conditions in the deeper layers), but also to the intense O_2_ depletion caused by polymorphonuclear leukocytes (PMNs), determining entire biofilm growth without aerobic respiration (29).

Downregulated DEGs also included the following: (i) two genes involved in flagellar biosynthesis (i.e., *fliF*, encoding the flagellar M-ring protein FliF, and *flhF*, encoding the flagellar biosynthesis protein FlhF); (ii) a NAD(P)H-quinone oxidoreductase protecting against ROS-induced oxidative stress, which was recently demonstrated to be part of the core biofilm transcriptome (PA1137) (30); and (iii) *nalD*, encoding a second repressor of the *mexAB-oprM* operon (31). Finally, consistent with previous studies on Pseudomonas response to zinc starvation, downregulation of *copA* and *copZ*, involved in copper efflux, was observed, suggesting interplay between zinc and copper homeostasis (Table 2) (32).

### NAC-mediated inhibition of P. aeruginosa denitrification pathway.

The role of NAC in the inhibition of the denitrification pathway was confirmed by measuring NO_3_^−^ and NO_2_^−^ concentrations during anaerobic growth of the P. aeruginosa Z154 strain (i.e., the strain used for transcriptomic analysis) in culture media supplemented with 10 mM NaNO_3_ or KNO_2_, in the presence or absence of NAC at 8,000 mg/L.

**FIG 6:**
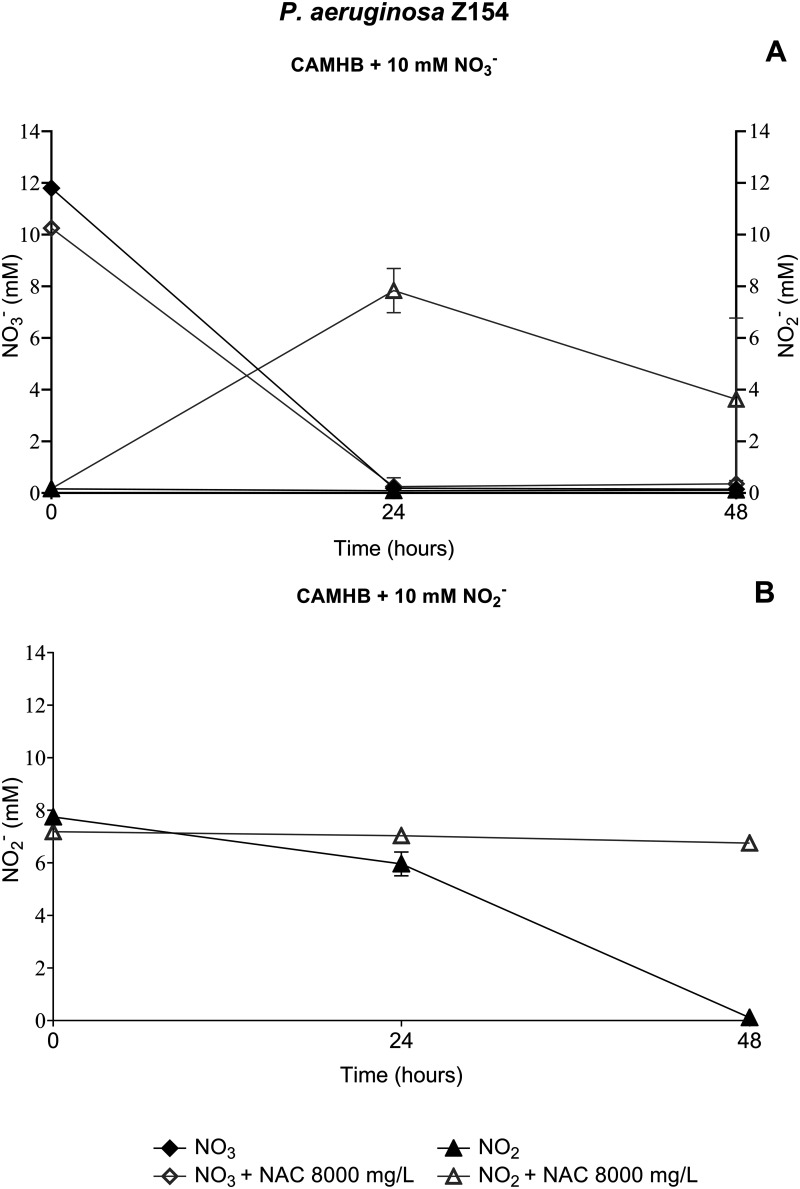
NAC-mediated inhibition of P. aeruginosa Z154 denitrification pathway. (A) NO_3_^−^ and NO_2_^−^ concentrations in anaerobic CAMHB supplemented with 10 mM NO_3_^−^, with or without NAC at 8,000 mg/L; (B) NO_2_^−^ concentration in anaerobic CAMHB supplemented with 10 mM NO_2_^−^, with or without NAC at 8,000 mg/L. Data are plotted as the mean values of NO_3_^−^ and/or NO_2_^−^ levels detected at each time point.

As expected from previous studies (33), in NaNO_3_-containing medium, the levels of NO_3_^−^ and its reduction product, NO_2_^−^, fell below the detection limit after 24 h, in the absence of NAC (Fig. 6A). However, in the presence of NAC at 8,000 mg/L, the depletion of NO_3_^−^ was followed by an accumulation of NO_2_^−^ (evident at both 24 and 48 h), indicating that further reduction of NO_2_^−^ was inhibited in the presence of NAC (Fig. 6A). In order to consolidate these data, the experiments were repeated using a medium supplemented with KNO_2_. In the absence on NAC, complete reduction of NO_2_ was observed after 48 h (Fig. 6B), as expected (33). On the contrary, in the presence of NAC at 8,000 mg/L, NO_2_ levels did not decrease (Fig. 6B).

These results were consistent with the transcriptomic data and showed that NAC was able to inhibit the denitrification pathway in anaerobic environments, such as those encountered in endobronchial CF mucus. This feature might contribute to the observed antibiofilm synergism of NAC-colistin combinations, as previously discussed.

### Time-kill assays of the NAC-colistin combination against planktonic cultures grown under anaerobic and aerobic conditions.

**FIG 7:**
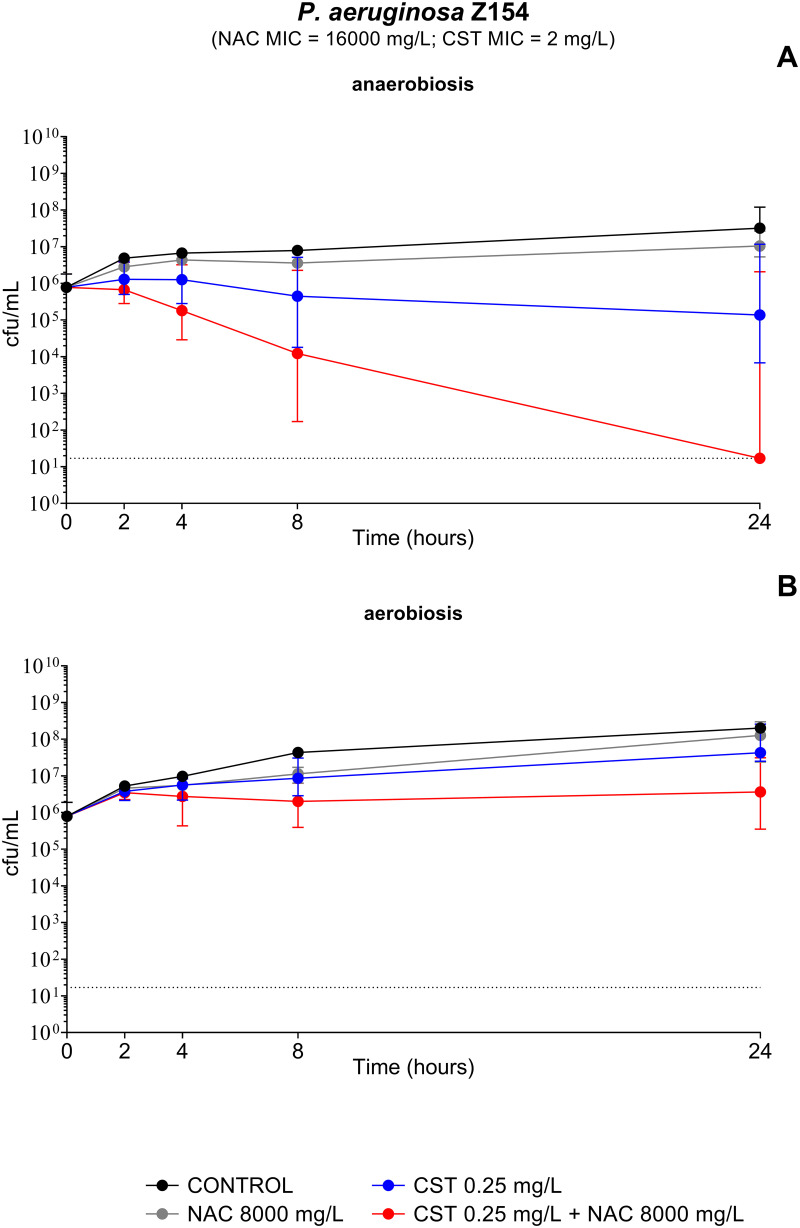
Time-kill curves of P. aeruginosa Z154 planktonic cultures exposed to *N*-acetylcysteine (NAC) at 8,000 mg/L, colistin (CST) at 0.25 mg/L, and the NAC-CST combination under anaerobic (A) and aerobic (B) conditions. NAC potentiated the bactericidal activity of colistin only under anaerobic conditions. Data are plotted as the median values of CFU per milliliter for each time point. Dotted lines indicate the detection limit (17 CFU/mL).

Transcriptomic and biological data from this study suggested a role of NAC in inhibiting the P. aeruginosa denitrification apparatus, which could contribute to the observed antibiofilm synergy of NAC-colistin combinations. In order to further investigate this issue, time-kill assays of the NAC-colistin combination were performed with P. aeruginosa Z154 (i.e., the strain used for transcriptomic analysis) planktonic cultures, under both anaerobic and aerobic conditions. Consistent with previous studies, anaerobic cultures were more susceptible to killing by colistin than aerobic cultures (34, 35) (Fig. 7A and B). Interestingly, a clear bactericidal effect of colistin at 0.25 mg/L (i.e., 1/8 MIC) in combination with NAC at 8,000 mg/L was observed in planktonic cultures grown under anaerobic conditions, with eradication achieved after 24 h of exposure (Fig. 7A). The wide error bars were due to the fact that in 2 out of 8 replicates (related to two independent experiments), no synergism was observed (Fig. 7A). This discrepancy was probably related to the low colistin concentration tested and the possible presence of heteroresistant subpopulations. On the contrary, cultures grown in the presence of oxygen were not affected by the NAC-colistin combination, demonstrating the influence of the growth conditions on the susceptibility of P. aeruginosa to such combination (Fig. 7B).

These results supported the hypothesis that, under anoxic conditions like those present in the deeper biofilm layers and in CF mucus, NAC-mediated inhibition of anaerobic respiration would prevent an adaptive response of P. aeruginosa to protect from colistin toxicity.

### NAC-mediated inhibition of P. aeruginosa swimming and swarming motility.

Transcriptomic results indicated that NAC downregulated two genes belonging to P. aeruginosa flagellar apparatus (i.e., *fliF* and *flhF*), which are necessary for the first step of flagellum assembly (36). In order to confirm the potential NAC-induced inhibition of flagellum-mediated motility, we performed classical swimming and swarming tests with the reference strain P. aeruginosa PAO1 and the CF strain P. aeruginosa Z154 (i.e., the strain used for transcriptomic analysis). P. aeruginosa Z154 was not capable of swarming motility under our laboratory conditions, so only the effect of NAC on swimming motility could be tested with this strain.

**FIG 8:**
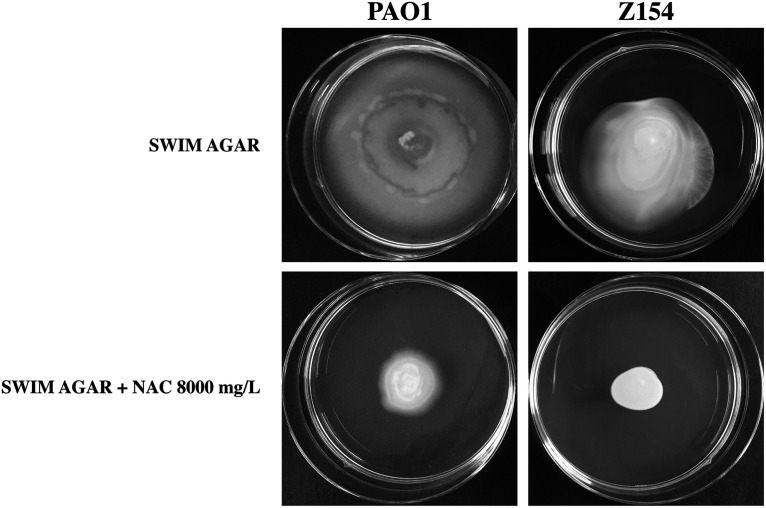
NAC-mediated inhibition of P. aeruginosa PAO1 and Z154 swimming motility. Assays were performed in at least three independent experiments (with three replicates per condition per experiment), and representative data are shown.

**FIG 9:**
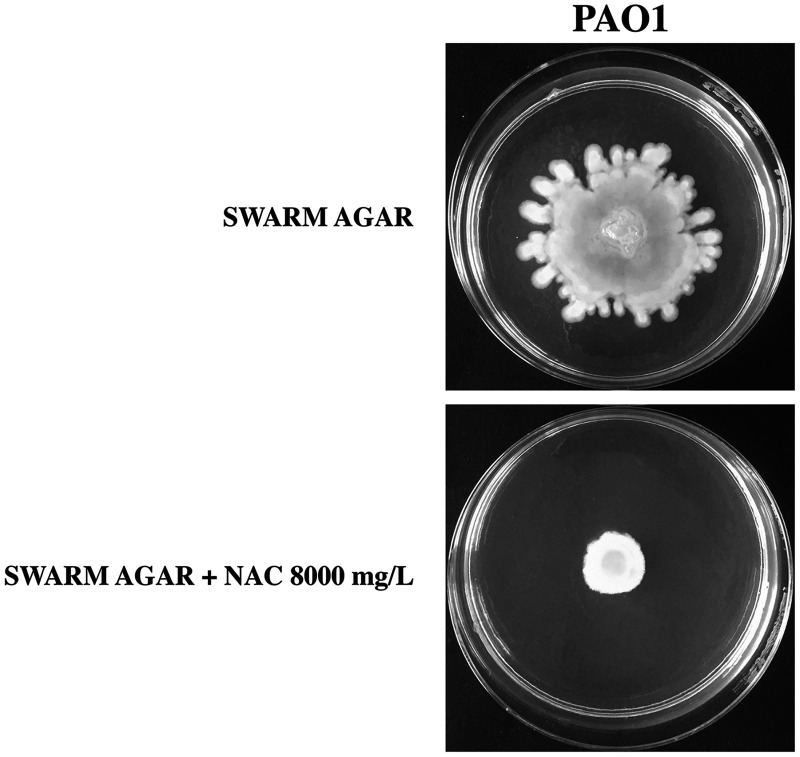
NAC-mediated inhibition of P. aeruginosa PAO1 swarming motility. Assays were performed in at least three independent experiments (with three replicates per condition per experiment), and representative data are shown.

Overall, the results showed a clear inhibition of both swimming and swarming motility in the presence of NAC at 8,000 mg/L (Fig. 8 and 9). Such inhibition could be related to the downregulation of crucial genes of the flagellar apparatus and/or the induction of a zinc starvation response. Indeed, zinc starvation has been demonstrated to affect the ability of P. aeruginosa to express several virulence phenotypes, crucial for the ability of this pathogen to colonize CF lung, including motility, biofilm formation and siderophore synthesis (37).

### Conclusions.

In conclusion, the results of this study demonstrated a relevant antibiofilm synergism of NAC-colistin combinations (at the high concentrations achievable by inhalation) against P. aeruginosa, which would deserve further investigation for potential clinical applications of inhaled formulations. Transcriptomic and biological experiments suggested that NAC inhibited P. aeruginosa anaerobic respiration, which could be relevant for the observed antibiofilm synergism with colistin.

In addition, although NAC alone was not demonstrated to be effective against preformed P. aeruginosa biofilms, transcriptomic analysis of NAC-exposed planktonic cultures revealed that NAC could attenuate P. aeruginosa virulence, mainly by inducing a zinc starvation response, affecting anaerobic respiration and inhibiting flagellum-mediated motility (with the last two features confirmed experimentally). In this perspective, NAC, at the high concentrations achievable by inhalation, might have beneficial effects in the very first steps of lung infection, possibly preventing biofilm formation and the establishment of a chronic colonization, which should be further investigated.

## MATERIALS AND METHODS

### Bacterial strains.

Seventeen strains were investigated, including 15 clinical isolates from CF patients, an MDR clinical isolate from a respiratory tract infection (RTI) from an intensive care unit (ICU), and the reference strain, P. aeruginosa PAO1 (Table 1). Identification was performed by matrix-assisted laser desorption ionization–time of flight mass spectrometry (MALDI-TOF MS) (Bruker, Shimadzu). Antimicrobial susceptibility was determined using the reference broth microdilution method (38). Whole-genome sequencing of clinical isolates was performed with the Illumina (San Diego, CA, USA) MiSeq platform, using a 2× 150-bp paired-end approach. Raw reads were assembled using SPAdes (39), and draft genomes were used to determine multilocus sequence types (MLSTs) and O types at the Oxford PubMLST site (https://pubmlst.org/) (40) and at the Center for Genomic Epidemiology site (https://cge.food.dtu.dk/services/PAst/) (41), respectively. The complete genome of P. aeruginosa Z154 was obtained by combining results from Illumina with those obtained using the Oxford Nanopore Technologies (Oxford, United Kingdom) MinION platform, and *de novo* assembly was generated using Unicycler v0.4.4 as previously described (42).

### Preparation of culture media.

NAC stock solutions (100 g/L) were prepared immediately before use. NAC powder (Zambon, Bresso, Italy) was dissolved in sterile distilled water, the pH was adjusted to 6.5 to 6.8 with NaOH at 10 M, and the solution was filtered through a 0.22-μm-pore membrane filter. All experiments were performed in cation-adjusted Mueller-Hinton broth (CAMHB) (Becton Dickinson, Milan, Italy), unless otherwise specified, starting from an appropriately concentrated medium to avoid broth dilution when NAC solution was used. The artificial sputum medium (ASM) was also used in selected experiments and was prepared as previously described by Kirchner et al. (43).

### *In vitro* biofilm susceptibility testing.

Biofilm susceptibility testing was first performed using the Nunc-TSP lid system (Thermo Fisher Scientific, Waltham, MA, USA), as described previously (44). Briefly, biofilms were grown for 24 h in CAMHB at 35°C under static conditions. Preformed biofilms were then exposed to NAC at 8,000 mg/L and colistin (colistin sulfate; Applichem, Darmstadt, Germany) at 2 to 32 mg/L, alone and in combination. The colistin concentration was selected according to preliminary results of antibiofilm susceptibility testing and the colistin MIC for each strain. After 24 h of exposure (i.e., 35°C, static conditions), biofilms were washed twice with 200 μL of phosphate-buffered saline (PBS) (Sigma-Aldrich, Milan, Italy) to remove loosely adherent bacteria, and sessile cells were removed from pegs by sonication for 30 min (Elma Transsonic T 460; Elma, Singen, Germany) in 200 μL of tryptic soy broth (TSB) (Oxoid, Milan, Italy) supplemented with 1% Tween 20 (Sigma-Aldrich) (i.e., the recovery medium). The median number of CFU per peg was then determined by plating 10 μL of appropriate dilutions of the recovery medium onto tryptic soy agar (TSA) (Oxoid) and incubating for 24 h at 35°C (detection limit, 20 CFU/peg). The colony count was also double-checked after 48 h of incubation.

The potential antibiofilm synergism of NAC-colistin combinations was further investigated using an *in vitro* ASM biofilm model (43) in order to mimic P. aeruginosa biofilm conditions within the CF mucus. The study was carried out with two selected CF strains (P. aeruginosa Z154 and Z34), exhibiting different features (i.e., mucoid/nonmucoid phenotype, antimicrobial susceptibility pattern, MLST, and O type) (Table 1). In brief, biofilms were grown in 2 mL ASM in 24-well plates (Sarstedt, Nümbrecht, Germany), for 72 h at 35°C under static conditions. Preformed biofilms were then exposed to NAC at 8,000 mg/L and colistin at 64 mg/L, alone and in combination. Preliminary experiments carried out with lower colistin concentrations (i.e., 2 to 32 mg/L) did not show evident synergistic antibiofilm activity, while higher colistin concentrations (i.e., >64 mg/L) led to eradication of the biofilm cultures even in the absence of NAC (data not shown). After 24 h of exposure (i.e., 35°C, static conditions), bacterial biofilms were disrupted by 30 min of sonication followed by manual pipetting, and the median number of CFU per milliliter was determined following the same protocol described for the Nunc-TSP lid assay.

Data from both biofilm models were obtained in at least three independent experiments, with at least 12 replicates per condition per experiment.

### RNA-seq and transcriptomic analysis.

P. aeruginosa Z154 (i.e., colistin-susceptible CF strain, mucoid, MDR, ST412, O6) (Table 1) was selected for studies aimed at investigating the transcriptomic response of P. aeruginosa to NAC exposure. A CF strain, rather than a reference strain (such as P. aeruginosa PAO1), was selected for this analysis because of the known adaptive diversification of P. aeruginosa into “specialized” types during chronic/recurrent infections in CF patients (3).

Because these represented the first data on the transcriptomic response of P. aeruginosa to NAC exposure, and considering the complex and still largely unknown effects of NAC on microbial physiology, we decided to perform the experiments with planktonic cultures, which represent a more homogenous and better standardized model for transcriptomic studies.

Overnight cultures in CAMHB were diluted at 1:50 in the same medium and incubated at 35°C with agitation to achieve an optical density at 600 nm (OD_600_) of 1.0. The cells were then exposed to NAC at 8,000 mg/L for 30 min at 35°C under static conditions. Cultures treated in the same way but not exposed to NAC represented the control. Total RNA extraction was performed using the SV total RNA isolation system (Promega, Madison, WI, USA) following the manufacturer’s instructions. rRNA depletion, cDNA library construction, and Illumina HiSeq 4000 platform-based transcriptome sequencing (RNA-seq) were performed by Eurofins Genomics Europe Sequencing (Constance, Germany). The transcriptome libraries were single-end sequenced with 50-bp reads for a total of 10 million reads per sample. Bioinformatic analysis was performed using the SeqMan NGen v17.3 software tool (DNASTAR Lasergene, Madison, WI, USA), with default parameters. Reads were aligned using P. aeruginosa Z154 complete genome (*n* = 6,344 coding DNA sequences [CDSs]) as a reference. Differentially expressed genes (DEGs) of the NAC-exposed cultures compared to the control were analyzed considering false-discovery rate (FDR) adjusted *P* values of <0.05 from DeSeq2. DEGs with a 99% confidence interval (CI) were discussed. Results were obtained from two independent experiments. In order to favor comparison with data present in the literature, genes without a univocal name have been indicated as P. aeruginosa PAO1 locus tags throughout the text and reported in Table 2 also as P. aeruginosa UCBPP-PA14 locus tags.

### NO_3_^−^ and NO_2_^−^ quantification.

NAC-mediated inhibition of the denitrification pathway was investigated by measuring the concentration of NO_3_^−^ and NO_2_^−^ in anaerobic cultures of P. aeruginosa Z154 (i.e., the strain used for transcriptomic analysis). For this purpose, the Griess nitrite/nitrate colorimetric assay (Cayman Chemicals, Ann Arbor, MI, USA) was used according to the manufacturer’s recommendations and as previously described, with some modification (33). CAMHB was supplemented with 10 mM NaNO_3_ or KNO_2_ and allowed to equilibrate for 3 days at 35°C in an anaerobic atmosphere by using the AnaeroGen kit (Oxoid). Overnight cultures were then diluted in 20 mL of each anoxic culture medium to reach a concentration of 10^6^ CFU/mL and challenged with NAC at 8,000 mg/L. At times 0, 24, and 48 h of incubation under anoxic conditions at 35°C, supernatants were harvested and subjected to Griess colorimetric reaction in order to detect NO_3_^−^ and NO_2_^−^ levels. NAC-free cultures represented the control. Experiments were carried out in triplicate with one replicate per time point per condition.

### Time-kill assays.

Time-kill assays were performed according to CLSI guidelines (45) with the colistin-susceptible strain P. aeruginosa Z154 (i.e., the strain used for transcriptomic analysis). Colistin at 0.25 mg/L was tested alone and in combination with NAC at 8,000 mg/L under both aerobic and anaerobic conditions. We decided to use this colistin concentration since a higher concentration led to eradication of the planktonic cultures (data not shown). The medium (CAMHB) used to obtain anoxic cultures was placed under an anaerobic atmosphere by using the AnaeroGen kit (Oxoid) for 3 days prior to use and during the whole experiment. The killing curves were carried out in borosilicate glass bottles with a final volume of 20 mL of CAMHB. At 0, 2, 4, 8, and 24 h of exposure, CFU per milliliter were determined by plating 60 µL of appropriate dilutions of each condition onto TSA and incubating for 24 h at 35°C (detection limit, 17 CFU/mL). Data were obtained from at least four independent experiments with two replicates per condition per experiment.

### Motility tests.

NAC-induced inhibition of flagellum-mediated motility (i.e., both swimming and swarming motility) was investigated with the reference strain P. aeruginosa PAO1, which has been used for similar motility experiments in several previous studies (46), and P. aeruginosa Z154 (i.e., the strain used for transcriptomic analysis). P. aeruginosa Z154 was not capable of swarming motility under our laboratory conditions (perhaps due to the known reduction of flagellar expression in mucoid CF-adapted strains) (47), so only the effect of NAC on swimming motility could be tested with this strain. Swim plates consisted of Luria-Bertani (LB) broth (Oxoid) containing 0.3% agar (46). Swarm plates consisted of nutrient broth (Oxoid) with 0.5% glucose and 0.5% agar (46). Overnight cultures in CAMHB were diluted in the same medium to a final OD_600_ of 3.0, and 5 μL was spotted onto swim and swarm plates, with or without NAC at 8,000 mg/L. Results were observed after incubation at 35°C for 48 h. Assays were performed in at least three independent experiments with three replicates per condition per experiment.

### Statistical analysis.

Statistical analysis of biofilm susceptibility assays was performed using GraphPad Prism version 8.0 (San Diego, CA, USA). Multiple-comparison tests were performed by the Kruskal-Wallis test with Dunn’s correction. A *P* value of ≤0.05 was considered significant. RNA-seq statistical analysis was performed using the SeqMan NGen v17.3 software tool.

### Data availability.

The complete genome sequence of P. aeruginosa Z154 was deposited in GenBank under accession no. CP069177. RNA-seq data were also deposited in the NCBI Gene Expression Omnibus (GEO) database under accession no. GSE190946.
